# Risk Adapted Ablative Radiotherapy After Intensive Chemotherapy for Locally Advanced Pancreatic Cancer

**DOI:** 10.3389/fonc.2021.662205

**Published:** 2021-04-20

**Authors:** Gabriella Rossi, Nicola Simoni, Salvatore Paiella, Roberto Rossi, Martina Venezia, Renato Micera, Giuseppe Malleo, Roberto Salvia, Tommaso Giuliani, Anthony Di Gioia, Alessandra Auriemma, Michele Milella, Stefania Guariglia, Carlo Cavedon, Claudio Bassi, Renzo Mazzarotto

**Affiliations:** ^1^ Department of Radiation Oncology, University of Verona Hospital Trust, Verona, Italy; ^2^ Department of General and Pancreatic Surgery, Pancreas Institute, University of Verona Hospital Trust, Verona, Italy; ^3^ Department of Oncology, Pancreas Institute, University of Verona Hospital Trust, Verona, Italy; ^4^ Department of Medical Physics, University of Verona Hospital Trust, Verona, Italy

**Keywords:** pancreatic cancer, locally advanced, SBRT (stereotactic body radiation therapy), hypofractionated ablative radiation, SAbR, ablative dose

## Abstract

**Background and Objective:**

To assess the efficacy of a Risk-Adapted Ablative Radiotherapy (RAdAR) approach, after intensive induction chemotherapy, in patients with locally advanced pancreatic cancer (LAPC).

**Material and Methods:**

Patients with LAPC who received RAdAR following induction chemotherapy from January 2017 to December 2019 were included in this observational study. The RAdAR approach consisted of an anatomy- and simultaneous integrated boost (SIB)-based dose prescription strategy. RAdAR was delivered with stereotactic ablative radiation therapy (SAbR), administering 30 Gy in 5 fractions to the tumor volume (PTV_t_) and 50 Gy SIB (BED_10_ 100 Gy) to the vascular involvement, or with (hypo-)fractionated ablative radiotherapy (HART) prescribing 50.4 Gy in 28 fractions to the PTV_t_, with a vascular SIB of 78.4 Gy (BED_10_ 100 Gy). Primary end points were freedom from local progression (FFLP), overall survival (OS), and progression-free survival (PFS).

**Results:**

Sixty-four LAPC patients were included.** **Induction chemotherapy consisted of gemcitabine/nab-paclitaxel in 60.9% and FOLFIRINOX in 39.1% of cases. SAbR was used in 52 (81.2%) patients, and HART in 12 (18.8%). After RAdAR, surgery was performed in 17 (26.6%) patients. Median follow-up was 16.1 months. Overall local control (LC) rate was 78.1%, with no difference between resected and non-resected patients (2-year FFLP 75.3% vs 56.4%; p = 0.112). Median OS and PFS were 29.7 months and 8.7 months, respectively, for the entire cohort. Resected patients had a better median OS (not reached versus 26.1 months; p = 0.0001) and PFS (19 versus 5.6 months; p < 0.0001) compared to non-resected patients. In non-resected patients, no significant difference was found between SAbR and HART for median FFLP (28.1 versus 18.5 months; p = 0.614), OS (27.4 versus 25.3 months; p = 0.624), and PFS (5.7 versus 4.3 months; p = 0.486). One patient (1.6%) experienced acute grade 4 gastro-intestinal bleeding. No other acute or late grade ≥ 3 toxicities were observed.

**Conclusions:**

The RAdAR approach, following intensive induction chemotherapy, is an effective radiation treatment strategy for selected LAPC patients, representing a promising therapeutic option in a multimodality treatment regimen.

## Introduction

The prognosis of pancreatic cancer remains dismal, with a 5-year survival rate of less than 10% (1, 2). Pancreatic cancer mortality continues to increase, and the advanced stage of disease at the time of diagnosis is predictive of decreased survival (3). At baseline staging, about 30-35% of patients present with non-metastatic, locally advanced pancreatic cancer (LAPC) and are not suitable for surgery (4). Chemotherapy represents the treatment of choice for LAPC, with consolidative radiotherapy as an option for patients in response to systemic therapy. However, overall survival remains unsatisfactory compared with patients eligible for surgical resection. In recent years, new more active multiagent chemotherapy regimens have changed this paradigm. In particular, gemcitabine plus nanoparticle albumin-bound (nab)-paclitaxel and 5-fluorouracil, leucovorin, irinotecan, plus oxaliplatin (FOLFIRINOX) combination regimens have been associated with improved oncological outcomes, thus becoming the standard of care (5–7).

LAPC is typically considered as a systemic condition, with the emergence of widespread metastatic disease representing the leading cause of mortality. Nevertheless, a John Hopkins rapid autoptic series showed that roughly one-third of patients died from locally destructive pancreatic cancer, regardless of systemic disease burden (8). Crane et al. found that isolated local disease progression leading to death occurred mainly in patients alive at more than 16 months, representing a significant cause of late disease-related mortality (9). Therefore, there is a strong rationale for using radiation therapy (RT), in combination with new multiagent chemotherapy regimens, to impact local control and potentially survival. Nonetheless, the use of standard-dose conventionally fractionated radiation therapy (CFRT), failed to demonstrate a significant improvement in survival duration of patients with unresectable pancreatic cancer (10). Thus, to improve oncological outcomes in LAPC, the use of more effective RT strategies is needed. In particular, the adoption of a higher than conventional biologically effective dose (BED) is advocated to achieve durable local tumor control and impact on survival (11).

In this context, stereotactic ablative radiation therapy (SAbR) and (hypo-)fractionated ablative radiotherapy (HART) have emerged as an effective component for the multimodal treatment of pancreatic cancer. Recent studies suggested that in LAPC the use of dose-escalated RT after induction systemic therapy can increase survival compared to either chemotherapy alone or CFRT (12–15). In addition, in the context of total neoadjuvant therapy, the combination of effective induction chemotherapy regimens followed by higher dose RT, might improve the likelihood of conversion to surgery also for LAPC patients (16, 17). However, for pancreatic tumors, the delivery of ablative doses is a challenge due to the proximity of extremely radiosensitive OARs, such as stomach, duodenum, and jejunum.

In the era of stereotactic techniques, advanced organ motion management, and accurate image guidance, two complementary approaches can be adopted to safely deliver ablative doses to the pancreatic tumor: the use of the simultaneous integrated boost (SIB) and the adoption of an anatomy-based dose prescription strategy. The SIB technique allows the simultaneous delivery of a differential dose to different target volumes during the same treatment fraction. The SIB may be used to increase the fraction (and total) dose and BED to the boost volume, without lengthening the overall treatment time. This dose-painted strategy may be used to administer ablative doses to the hypoxic center of the pancreatic tumor, while the target area near critical OARs is covered by a safe dose (18). The anatomy-based dose prescription strategy involves the radiobiological principle of fractionation (19). Supposed ablative doses cannot be safely administered with an extremely hypofractionated regimen (5 fractions SAbR), due to an unacceptable risk of toxicity. In this case, the solution is to increase the number of fractions, incorporating a dose painted ablative SIB into a conventional 28-fraction RT course (HART). With HART, it is possible to reduce the BED to OARs, while maintaining higher effective doses inside the tumor. The RT strategy, based on these dose prescription principles, is the Risk Adapted Ablative Radiotherapy (RAdAR) approach.

The aim of this retrospective study was to evaluate the impact of the RAdAR approach on local control, survival, disease progression, and toxicity, in patients with LAPC previously treated with intensive induction chemotherapy.

## Materials and Methods

### Patient Characteristics

This study is an observational single-center analysis of prospectively collected data, designed to assess the safety and effectiveness of the Risk Adapted Ablative Radiotherapy (RAdAR) approach in patients with LAPC. The Institutional Review Board (IRB) approved the prospective collection of data (PAD-R n.1101 CESC). LAPC patients, candidate to radiotherapy following induction chemotherapy by the Verona Pancreas Institute Oncologic Multidisciplinary Group from January 2017 to December 2019, were included. The institutional pancreatic cancer management pathway has already been described elsewhere (7). The National Comprehensive Cancer Network (NCCN) classification was used to define the tumor as locally advanced at the time of diagnosis (**Table S1 Supplementary Material**) (20). The following data were prospectively collected: baseline demographics, diagnostic work-up, and tumor characteristics, including tumor size, major vessels involvement, presence of enlarged (short axis > 10 mm) regional nodes, and involvement of the gastrointestinal tract (duodenum/stomach). Data on chemotherapy regimens, Ca 19-9 value, radiation therapy modalities, treatment toxicities, surgical outcomes and postoperative complications, and tumor pathology characteristics were obtained.

Induction chemotherapy primarily consisted of combination regimens with gemcitabine plus nanoparticle albumin-bound (nab)-paclitaxel or 5-fluorouracil, leucovorin, irinotecan, plus oxaliplatin (FOLFIRINOX), according to the treating oncologist’s discretion. In the absence of disease progression at restaging, radiation therapy was offered after multidisciplinary discussion regardless of the chemotherapy administered. Exclusion criteria for the RAdAR approach were: an ECOG performance status ≥ 2, less than 3 months of systemic therapy administration, biochemical Ca19-9 increase or loco-regional progression disease at CT-scan after induction chemotherapy, metastatic disease, patients treated for recurrent disease after resection, and/or prior radiation therapy to upper abdomen.

### Radiation Therapy Protocol

Details of the radiation treatment have previously been reported (16). Briefly, most patients had fiducial markers (3 to 4 gold seeds) placed around the pancreatic tumor by endoultrasonography (EUS) before simulation CT. Instead in others, a surrogate structure, such as a biliary stent, was used for daily image guidance. Patients were immobilized in a supine position with arms over the head. A custom-made Vac-Lok™ cushion and abdominal compressor (Body Pro-Lok™, Civco, Coralville, IA) were used to reduce breathing-induced tumor motion. A tri-phase contrast-enhanced simulation CT was performed after a scan without contrast. An integrated gross tumor volume (iGTV) was defined as the envelope of the GTVs delineated on each CT phase. An iGTV-to-PTV margin of 5 mm was applied to generate the PTV tumor (PTV_t_). A PTV high dose (PTV_hd_) was generated to encompass the vascular involvement, including the entire tumor-vessel circumferential interface (TVI) and major vessels encasement, with a 5 mm expansion. Elective nodes (elective nodal irradiation, ENI) were not included in the treatment volume.

**Figure 1:**
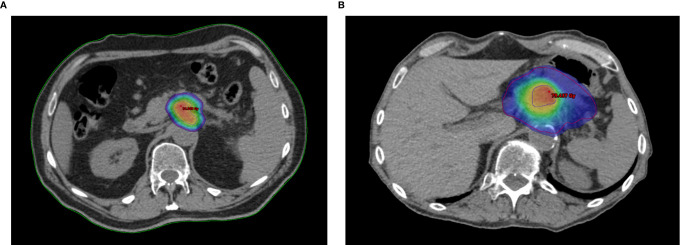
Example plans of Risk-Adapted Ablative Radiotherapy (RAdAR) approach in LAPC. Dose distribution for Stereotactic ablative radiation therapy (SAbR) **(A)** and (hypo-)fractionated ablative radiotherapy (HART) **(B)**. The isodoses are highlighted with color wash set at 95% of the PTV_t_ prescription dose (30 Gy/5 fractions for SAbR and 50.4 Gy/28 fractions for HART). As shows in figure, an ablative SIB (BED_10_ = 100 Gy) within the tumor is prescribed for both SAbR (50 Gy/5 fractions) and HART (78.4 Gy/28 fractions). The PTV_t_ in shown in red, and the PTV_hd_ in blue.

The RAdAR approach consisted of an anatomy- and SIB-based dose prescription strategy. If anatomically and dosimetrically feasible, the first treatment choice was the stereotactic ablative radiation therapy (SAbR). Instead, the (hypo-)fractionated ablative radiotherapy (HART) schedule was adopted in the following cases: tumor ≥ 6 cm in greatest dimension, nodal spread of disease that could not be included in the SAbR target volume, tumor adhesion/infiltration of the stomach or duodenum, and/or impossibility to achieve SAbR planning objectives (e.g., non-respect of OARs dose constraints). An example of SAbR and HART plans is showed in **Figure 1**.

SAbR was delivered in 5 consecutive daily fractions, prescribing 30 Gy to the PTV_t_, while simultaneously delivering a 50 Gy SIB (BED_10_ 100 Gy) to the PTV_hd_. If necessary, the prescribed dose was reduced to 25 Gy on the overlap area between the PTV_t_ and the planning organ at risk volume (OARs + 3 mm expansion), using a simultaneous integrated protection (PTV_sip_) approach (21). Normal tissue constraints were as follows: for duodenum, bowel and stomach Dmax < 35 Gy, V30 Gy < 5 cc, Dmean < 20 Gy; for spinal cord Dmax < 20 Gy; for kidneys Dmean < 10 Gy and D200cc < 17.5 Gy; and for liver D700cc < 21 Gy. For the HART schedule, the dose prescription was 50.4 Gy in 28 fractions to the PTV_t_, while simultaneously delivering a 78.4 Gy SIB (BED_10_ 100 Gy) to the PTV_hd_. As described by Crane, a Dmax < 60 Gy for the stomach and descending duodenum was adopted, while for the transverse duodenum and jejunum (out of reach of an endoscopic haemostatic procedure), a Dmax < 54 Gy was used as a precaution (11). Normal tissue constraints commonly used for CFRT were adopted for other OARs. No concomitant chemotherapy was prescribed during SAbR, while for HART, Gemcitabine or Capecitabine were concurrently used for tumor radiosensitization.

Coverage goals for PTV targets in both SAbR and HART schedule were D_98_≥95% for PTV_hd,_ a maximum point dose of 110% inside PTV_hd_, D_95_≥95% for PTV_t_ (and PTV_sip_ for SAbR). The RAdAR was delivered using RapidArc^®^ Technology (Varian Medical Systems, Palo Alto, CA) or TomoTherapy^®^ System (Accuray, Sunnyvale, CA). Daily on-line volumetric image-guided radiotherapy (cone beam or megavoltage CT) was performed before each treatment fraction. A 3-hour fasting period prior to simulation CT and before each radiation therapy fraction was required, to reduce inter- and intra-fractional variability.

### Response Evaluation and Follow-Up

Restaging CT scans were performed within 4-6 weeks from the end of the RAdAR. Response evaluation was performed using response evaluation criteria in solid tumors (RECIST) (22). Patients were re-evaluated by a multidisciplinary team, and, in the absence of tumor progression, re-considered for surgery. If a radical resection was not considered feasible, patients were deemed candidate for follow-up. Follow-up examinations were then performed every 3 months after completion of RT or surgical resection for resected cases. The follow-up schedule included a thorax/abdomen contrast-enhanced CT-scan, blood chemistries including Ca 19-9 and CEA markers, and a clinical assessment. RT acute and late toxicity data were collected during the follow-up according to common terminology criteria for adverse events (CTCAE) version 4.0 (23). Acute toxicity was defined as occurring within 90 days from RT completion, while late toxicity was defined as occurring > 90 days after RT. The disease failure site was documented as loco-regional (LRF), systemic, or mixed.

### Statistical Analysis

Quantitative variables were described as median and interquartile range (IQR) or mean and standard deviation (SD), categorical variables were summarized as counts and percentages. The primary endpoints were local control (LC), overall survival (OS) and progression-free survival (PFS). The secondary endpoint was toxicity. Freedom from local progression (FFLP) was calculated from the end of RT to the first assessment of loco-regional progression, as per RECIST criteria. OS was defined as the time from the diagnosis to death; PFS was calculated from RT to the date of a documented disease progression, relapse, or death. Patients who did not develop an event during the study period were censored at the date of last observation. For the survival analysis, patients surgically explored but not resected were integrated into the non-resected group. The survival probabilities were estimated using the Kaplan-Meier method and reported with their 95% confidence interval (CI). Comparisons among strata were performed using the log-rank test. Statistical analyses were performed using SPSS software version 25 (IBM, Chicago, IL) and MedCalc Statistical Software version 16.4.3 (MedCalc Software bv, Ostend, Belgium). A *p* value < 0.05 was considered statistically significant.

## Results

### Baseline Characteristics

Baseline characteristics are outlined in **Table 1**. A total of 64 LAPC patients were included in the analysis; of these 36 (56.3%) were male. Median age was 65.8 years (IQR 58.5-70.4 years). The median number of induction chemotherapy cycles was 6 for gemcitabine/nab-paclitaxel (IQR 6-6) and 12 for FOLFIRINOX (IQR 10-12). Following primary chemotherapy, 26 (40.6%) patients had a partial response, and 38 (59.4%) a stable disease as per RECIST criteria.

**Table 1:** Baseline characteristics and treatment details.

No. of patients	64
Age, years, median (IQR)	65.8 (58.5-70.4)
Sex, male, n (%)	36 (56.3)
ECOG, 0, n (%)	55 (85.9)
Primary tumor location, n (%)	
- Head - Body - Neck	39 (60.9) 23 (35.9) 2 (3.2)
Tumor size (mm), median (IQR)	40 (32-45)
Biliary stent, present, n (%)	22 (34.4)
CA19-9 (U/mL) at diagnosis, mean (SD)	1000 (±1168)
CA19-9 (U/mL) after chemotherapy (before RAdAR), mean (SD)	109 (±162)
Clinical T stage*, n (%)	
- T2 - T3 - T4	6 (9.4) 11 (17.2) 47 (73.4)
Clinical N stage*, n (%)	
- N0 - N1 - N2	30 (46.9) 32 (50) 2 (3.1)
Vascular involvement, n (%)	
- CA - SMA - PV - SMV - CHA	30 (46.9) 32 (50) 24 (37.5) 22 (34.4) 16 (25)
Pre-RAdAR chemotherapy regimen, n (%)	
- FOLFIRINOX - Gemcitabine + nab-paclitaxel	25 (39) 39 (61)
RAdAR technique, n (%)	
- SAbR - HART	52 (81.2) 12 (18.8)
Delivery technique, n (%)	
- RapidArc^®^ Technology - TomoTherapy^®^ System	48 (75) 16 (25)

IQR, interquartile range; SD, standard deviation; CA, celiac artery; SMA, superior mesenteric artery; PV, portal vein; SMV, superior mesenteric vein; CHA, common hepatic artery; FOLFIRINOX, fluorouracil, leucovorin, oxaliplatin; SAbR, stereotactic ablative radiation therapy; HART, hypofractionated ablative radiotherapy.

*Per the AJCC staging system, eighth edition.

### RAdAR Protocol Data

Median GTV, PTV_t_ and PTV_hd_ were 24.4 cc (IQR 15.2-35.5 cc), 70.1 cc (IQR 50.2-94.6 cc), and 18.1 cc (IQR 14.7-22.6 cc), respectively. For SAbR the median value for the mean GTV dose was 44.2 Gy (IQR 41.1-47.8 Gy), corresponding to a median GTV BED_10_ of 83.3 Gy, while for HART was 65.6 Gy (IQR 55.3-68.2 Gy), for a median GTV BED_10_ of 81 Gy.

The RAdAR was delivered after a median of 5 weeks (IQR 4-6 weeks) from the end of chemotherapy. The SAbR was used in 52 (81.2%) patients, while HART was the treatment of choice in the remaining 12 (18.8%). All patients completed the prescribed treatment. At first restaging after RT, a partial response was observed in 11 (17.2%) patients, while a disease progression in 2 (3.1%). In the remaining patients, the disease was stable as per RECIST criteria.

RT-related acute adverse events of any grade were reported in 26 (39.1%) patients. The most frequent acute symptoms were grade 1 or 2 fatigue (45.3%), abdominal pain (37.5%) and/or nausea (30.3%). One patient (1.6%) experienced a grade 4 duodenal bleeding within 3 months after the end of HART, which was successfully treated with endoscopy. Remarkably, no late grade ≥ 3 toxicities were registered. **Table 2** outlines the most common acute adverse events reported.

**Table 2:** RAdAR-related acute toxicity.

	Grade 1	Grade 2	Grade 3	Grade 4	Grade 5
**Fatigue, n (%)**	20 (31.2)	9 (14.1)	0 (0)	0 (0)	0 (0)
**Anorexia, n (%)**	9 (14.1)	4 (6.2)	0 (0)	0 (0)	0 (0)
**Dyspepsia, n (%)**	17 (26.6)	5 (7.8)	0 (0)	0 (0)	0 (0)
**Nausea, n (%)**	16 (14.7)	10 (15.6)	0 (0)	0 (0)	0 (0)
**Vomiting, n (%)**	3 (4.7)	1 (1.6)	0 (0)	0 (0)	0 (0)
**Diarrhea, n (%)**	5 (7.8)	4 (6.2)	0 (0)	0 (0)	0 (0)
Abdominal pain*, n (%)	14 (21.9)	10 (15.6)	0 (0)	0 (0)	0 (0)
**GI bleeding, n (%)**	0 (0)	0 (0)	0 (0)	1 (1.6)	0 (0)

RAdAR, Risk Adapted Ablative Radiotherapy; GI, gastro-intestinal.

*appearance or worsening.

### Surgical Data

Twenty-seven patients (42.2%) were deemed eligible for exploratory laparotomy after RT, and tumor resection was achieved in 17 (26.6%) patients, with a resection/exploration ratio of 63%. For the remaining 10 patients, the resection was aborted due to intraoperative findings of extensive local tumor infiltration in 6 (22.2%), and liver metastases in 4 (14.8%). Surgery was carried out at a median of 5 weeks (IQR 4-6 weeks) from the end of RT. An R0 resection was achieved in 11 (64.7%) patients, and 1 (1.6%) patient showed a complete pathologic response (**Table S2 Supplementary Materials**). Postoperative gastric emptying, clinically relevant pancreatic fistula, and post-pancreatectomy hemorrhage, occurred in 7.8%, 6.2%, and 4.7% of resected patients, respectively. The 90-day mortality was nil.

### Survival and Pattern of Relapse

**Figure 2:**
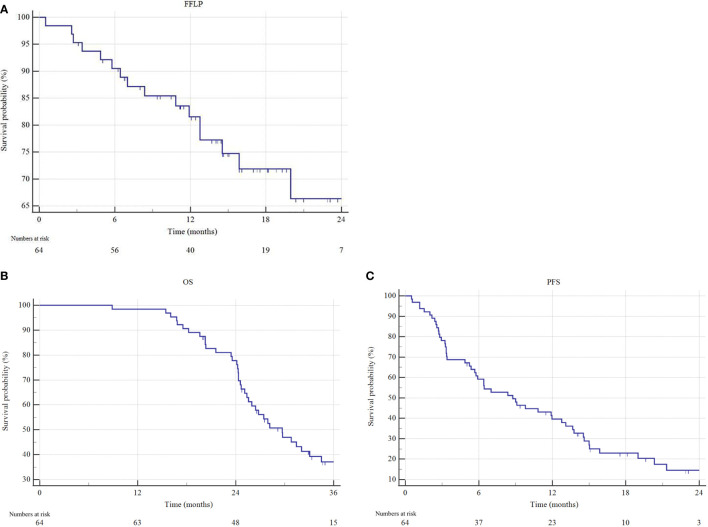
Freedom from local progression (FFLP), overall survival (OS) and progression free survival (PFS) estimated by Kaplan–Meier method. **(A)** FFLP, **(B)** OS and **(C)** PFS of the entire cohort.

The median follow-up for the analysis was 16.1 months (IQR 11.5-21 months) in the overall cohort and 22.9 months (IQR 17.7-30.8 months) among patients still alive at the last observation. The overall local control (LC) rate was 78.1%, with a 2-year FFLP rate of 66.1%. At the last follow-up, 27 (42.2%) patients were alive, and 10 (15.6%) were disease-free. For the entire cohort, the median OS was 29.7 months (95% CI 25.4-34.4) and the median PFS was 8.7 months (95% CI 5.7-12.8) (**Figure 2**).

**Figure 3:**
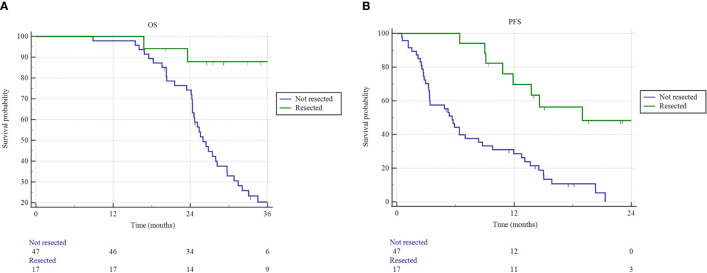
Overall survival (OS) and progression free survival (PFS) estimated by Kaplan–Meier method. **(A)** OS and **(B)** PFS as a function of resection status (resected versus not resected patients).

The median OS for resected patients has not been reached, compared to 26.1 months (95% CI 24.3-29.7) of non-resected patients (p = 0.0001) (**Figure 3**). Similarly, the median PFS of resected patients was 19.0 months (95% CI 10.8-19.5), versus 5.6 months (95% CI 3.3-21.3) of non-resected patients (p < 0.0001). One- and 2-year freedom from local progression (FFLP) did not significantly differ between the 2 groups, being 87.8% and 75.3% versus 82.1% and 56.4%, in resected versus non-resected patients, respectively (p = 0.112).

In non-resected patients, no significant difference was found between SAbR and HART for median FFLP (28.1 versus 18.5 months; p = 0.614), OS (27.4 versus 25.3 months; p = 0.624), and PFS (5.7 versus 4.3 months; p = 0.486). Similarly, in the non-resected cohort, the chemotherapy regimen adopted did not impact on survival (median OS 25.6 months for gemcitabine/nab-paclitaxel versus 26.8 months for FOLFIRINOX; p = 0.298).

Tumor recurrence was observed in 50 (78.1%) patients, with a systemic, loco-regional, and mixed pattern in 36 (56.2%), 6 (9.3%), and 8 (12.5%) respectively. No isolated regional recurrence occurred in the ENI area. Overall, the cumulative incidence of disease failure was 58.8% in resected patients versus 85.1% in non-resected patients (p=0.025).

## Discussion

In the era of multimodal therapy for LAPC, based on multiagent chemotherapy regimens, and radiotherapy, this observational study reports the efficacy and safety of a novel Risk-Adapted Ablative Radiotherapy (RAdAR) approach. To the best of our knowledge, this study includes the first cohort of LAPC patients treated with intensive induction chemotherapy, followed by an anatomy- and SIB-based ablative radiotherapy dose-prescription strategy, using SAbR and HART.

LAPC is one of the most common diagnoses of pancreatic cancer. Historically, LAPC has had poor prognosis and palliative monochemotherapy regimens (e.g., gemcitabine alone) were the only suitable treatment, leading to a median survival of 6 months (24). The addition of conventionally fractionated radiation therapy (CFRT) to induction monochemotherapy has led to a modest impact on long-term tumor control and survival (10, 25–27). Recently, multiagent chemotherapy regimens (gemcitabine/nab-paclitaxel and FOLFIRINOX) have proved to almost double the survival of LAPC compared to monochemotherapy schedules (5, 7). In particular, a recent meta-analysis by Suker et al. reported a median OS of 24.2 months for selected LAPC patients treated with FOLFIRINOX (6). Thus, a new frontier in pancreatic cancer research is the clinical evaluation of the combination of these intensive chemotherapy regimens with ablative radiation therapy approaches (SAbR and HART), to maximize the oncological benefits.

In the RAdAR approach, ablative BED prescription was based on two key principles: the SIB and the anatomy-based prescription dose. A SIB dose escalation to the hypoxic center of the pancreatic tumor is currently advocated to improve oncological outcomes in pancreatic cancer (11). Promising data in terms of the effectiveness of SIB use in LAPC have been described with different RT schedules (12, 16, 28–30). A retrospective study evaluating 200 patients with LAPC found that patients receiving a dose-escalated RT with a tumor burden SIB in 15-28 fractions had improved oncological outcomes compared with CFRT (12). Patients who received BED > 70 Gy had a superior OS (17.8 vs 15.0 months, p = 0.03), with a 2-year OS rates of 36%, and local-regional recurrence-free survival (10.2 months versus 6.2 months for BED < 70 Gy, p = 0.05). Stereotactic body radiotherapy (SBRT) SIB approach, delivered in 5 fractions with a median total dose of 30 Gy to the tumor and 40 Gy dose painted to the TVI, was described by Mellon et al. (28). Median OS for LAPC patients was 15.0 months and increased to 34.2 months in resected patients, with a 1-year local control rate of 78% for patients not undergoing resection. In the present study, an ablative SIB (BED_10_ = 100 Gy) within the tumor was prescribed for both SAbR (50 Gy in 5 fractions) and HART (78.4 Gy in 28 fractions). With a median follow-up of 16.1 months (22.9 months in censored patients), the median OS and PFS were 29.7 and 8.7 months, respectively. In addition, as a consequence of the high dose gradient within the tumor target related to the SIB use, the median value for the mean GTV dose was 44.2 Gy for SAbR and 65.6 Gy for HART, corresponding to a median GTV BED_10_ of 83.3 Gy and 81 Gy, respectively. Thus, the tumor absorbed dose resulted consistently higher than the one prescribed to the PTV_t_, increasing the final local effect of the RAdAR. Indeed, the 2-year freedom-from-local-progression (FFLP) in our study did not significantly differ between resected versus non-resected patients (81.6% versus 56.4%; p = 0.112).

Simultaneously, a risk-adapted approach was used to administer these ablative doses regardless of tumor characteristics. In particular, the HART was adopted in 6 (9.4%) cases for tumor ≥ 6 cm in greatest dimension or nodal spread of disease that could not be included in the SAbR target volume, in 5 (7.8%) patients due to tumor adhesion/infiltration of the stomach or duodenum, and in 1 (1.6%) due to the impossibility to achieve the SAbR planning objectives. Interestingly, in non-resected patients HART was not inferior, when compared to SAbR, for median FFLP (28.1 versus 18.5 months; p = 0.614), OS (27.4 versus 25.3 months; p = 0.624), and PFS (5.7 versus 4.3 months; p = 0.486). This confirms the importance of administering ablative doses to the pancreatic tumor, regardless of the schedule adopted. Moreover, the RAdAR approach, using extremely accurate delivering techniques, advanced organ motion management, and daily image guidance, resulted well tolerated, with a single (1.6%) case of grade ≥ 3 of RT-related toxicity.

In the present study, median OS of non-resected patients was 26.1 months, likely superior to those reported in historical CFRT and SBRT series (31). Undoubtedly, our study included a highly selected cohort of LAPCs. In particular, patients strongly pre-treated with chemotherapy, for a median period of 6 months, were selected for RAdAR; instead, patients with disease progression (radiological and/or biochemical) after systemic therapy, were excluded. Thus, it could be postulated that in patients who achieve a good response to chemotherapy, the addition of RT at non-standard doses may improve oncological outcomes. This indication has also been suggested by other Authors. In a recent retrospective analysis of 149 LAPC treated with intensified chemotherapy and SAbR, the combination of SAbR doses ≥ 40 Gy and FOLFIRINOX was associated with a median OS of 24 months (15). Similarly, the results of the Memorial Sloan Kettering Cancer Center network ablative LAPC program showed an impressive median OS of 27 months from diagnosis and a 2year-FFLP of 54% from RT, in 136 LAPC patients with a median follow-up of 16 months (32). Remarkably, patients were treated with ablative doses, with different fractionations (75 Gy/25 fractions, 67.5 Gy/15 fractions, or 50 Gy/5 fractions) according to the distance between pancreatic tumor and the gastro-intestinal tract. In addition, in a more recent prospective trial of SAbR (30-45 Gy/3 fractions) for unresectable pancreatic cancer, the Authors found a median survival of 23 months from the time of diagnosis (33). Notably, patients were enrolled regardless of the number of cycles and systemic therapy schedule, emphasizing the synergistic potential role of the ablative RT doses.

In addition to the aforementioned motivations, other factors potentially contributed to the encouraging survival data of the present study. At our Institution we adopt a follow-up policy including active surveillance at tight time intervals rather than symptoms-oriented surveillance. Thus, all patients included in the analysis underwent a rigorous follow-up, with CT scans, Ca 19.9 and clinical evaluation every 3 months after RT in the first year. This allowed for the early detection of disease recurrence (radiological and/or biochemical), and prompt first line chemotherapy initiation. Indeed, 73.4% of patients were able to start systemic therapy at the time of disease recurrence, potentially contributing to the clinical outcomes.

In the retrospective series by Toesca et al., the use of FOLFIRINOX was superior in terms of OS and PFS compared to regimens that utilize gemcitabine-based chemotherapy, followed by SAbR (15). Instead, in our series, the survival impact of FOLFIRINOX compared to gemcitabine/nab-paclitaxel was not significant. In particular, in the non-resected cohort, median OS was 25.6 months for gemcitabine/nab-paclitaxel versus 26.8 months for FOLFIRINOX (p = 0.298). However, this result should be interpreted with caution, considering that the choice of the chemotherapy schedule was not standardized and at the discretion of the referring oncologist. Therefore, definitive conclusions cannot be drawn.

In this highly selected patient cohort of LAPC, the effect of surgery was remarkable. The surgery group had a 2-year OS and PFS rate of 87.8% and 48.4%, respectively. At the last follow-up, 88.2% of the resected patients were alive. This conclusion coincides with previous findings reported in the literature. Indeed, Gemenetzis et al. found that surgical resection of LAPC after neoadjuvant therapy was associated with a median OS of 35.3 months (34). Similarly, Murphy et al. reported that the combination of FOLFIRINOX and radiotherapy provided a high rate of LAPC downstaging, with an 86% surgical resection rate, and a 2-year OS and PFS of 82.9% and 44.2%, respectively (17). The possibility of increasing the resection rate in LAPC patients by means of current neoadjuvant systemic therapies, followed by radiotherapy strategy with ablative boost doses to the vessels involvement, is an intriguing strategy to be validate in further studies.

A further critical aspect of pancreatic cancer radiotherapy is represented by the definition of target volumes. The RAdAR approach is based on the following considerations: (1) as a consequence of the dose inhomogeneity within the PTV_t_ related to the SIB use, the median BED_10_ received by the GTV was > 80 Gy, a dose presumed to be necessary to optimize local control (35); (2) the administration of ablative doses to the tumor-vessel interface (TVI), can sterilize the tumor boundaries encasing the pancreatic vessels, potentially allowing a conversion to surgery; and (3) the use of safe prescription doses to the PTV_t_ can reduce the risk of RT-related serious adverse events (e.g. late toxicity). However, there is great variability between Authors in the definition of target (and SIB) volumes in LAPC (12, 15–17, 28–30, 32–34). The advovated benefit of prescribing an ablative dose to the PTV_t_ to improve oncological outcomes, define a clinical target at the Authors’ Institution (36).

Radiation therapy for pancreatic cancer is rapidly evolving. Key elements for administering ablative doses in such a complex context are organ motion management and the possibility to optimize treatment plan dose distribution based on daily tumor and OAR anatomy. Currently, early experiences with the use of Magnetic Resonance-guided Radiation Therapy (MRgRT) have been described, with promising results (37, 38). The ability of MRgRT to perform high quality on board imaging, a “real-time” MR gating, as well as on-line adaptive RT before each fraction delivery, are undoubtedly attractive. Rudra et al. reported a 2-year OS of 67% in patients treated with BED > 70 Gy using MRgRT, with an excellent toxicity profile (37). In addition, Hassanzadeh and Colleagues, using a MR online adaptation strategy, found that in an unoptimized plan approach, duodenum dose constraints would have been violated in 67.7% of fractions (38). Overall, MRgRT could allow for further, safer, dose escalation in LAPC. However, if the use of this technology might translate in superior clinical results, compared to current standard Linac RT approaches (with volumetric modulated RT, volumetric image-guidance, and advanced organ motion management), is still an unanswered question.

Our study is limited due to its retrospective, single-institution design and the relatively small sample size. Moreover, our results could be affected by the patient selection process since the indication to RAdAR was defined on a case-by-case basis by our multidisciplinary board, reflecting the distinctive characteristics of our practice. Finally, the decision for surgical exploration was at the surgeon’s discretion, adding a selection bias and heterogeneity to the outcomes measured.

In conclusion, the RAdAR approach, following intensive induction chemotherapy, is an effective radiation treatment strategy for selected LAPC patients, representing a promising therapeutic option in a multimodality treatment regimen. This anatomy- and simultaneous integrated boost (SIB)-based dose prescription strategy enhanced the delivery of ablative biologically effective doses to the tumor, with an excellent toxicity profile. Further studies are needed to corroborate these results.

## Data Availability Statement

The datasets presented in this article are not readily available because the datasets generated for this study are available on request to the corresponding author, subject to approval by the Institutional Review Board. Requests to access the datasets should be directed to nicolasimoni81@gmail.com.

## Ethics Statement

The studies involving human participants were reviewed and approved by Comitato etico per la Sperimentazione Clinica (CESC) delle Province di Verona e Rovigo. The patients/participants provided their written informed consent to participate in this study.

## Author Contributions

Conceptualization: GR and NS. Methodology: NS and SP. Software: SP. Validation: RS, MM, and RMa. Formal analysis: GR, NS, and SP. Data curation: MV, RR, GM, TG, AG, and AA. Writing—original draft preparation: GR, NS, SP, and SG. Writing—review and editing: MV, RR, RMi, and GM. Images curation: GR and SG. Supervision: RS, MM, CC, CB, and RMa. All authors contributed to the article and approved the submitted version.

## Conflict of Interest

The authors declare that the research was conducted in the absence of any commercial or financial relationships that could be construed as a potential conflict of interest.
