# Supplementary material for: Risk Adapted Ablative Radiotherapy After Intensive Chemotherapy for Locally Advanced Pancreatic Cancer
Source: Front Oncol. 2021 Apr 20;11:662205. doi: 10.3389/fonc.2021.662205 (PMC8093383; doi:10.3389/fonc.2021.662205)
Supplement: Supplementary file 1 [file Table_1.docx]

Supplementary Material

**Table Supplementary1**. Criteria for defining locally advanced pancreatic cancer (LAPC)

| **Unresectable locally advanced disease** |
| --- |
| Tumors considered locally advanced include the following:   - Unreconstructible superior mesenteric vein or portal vein occlusion - Greater than 180 degrees superior mesenteric artery or celiac artery encasement - Aortic invasion |

Adapted from Network NCC Pancreatic Adenocarcinoma Version 2.2015. NCCN Guidelines. (2015).

**Table Supplementary2**. Surgical data of resected patients (n=17)

| Surgical procedure, n (%)   - Pancreaticoduodenectomy - Distal pancreatectomy - Total pancreatectomy | 8 (47.1)  6 (35.3)  3 (17.6) |
| --- | --- |
| Vascular resection, yes, n (%) | 7 (41.2) |
| Pathological T (pT) stage*, n (%)   - T0 - T1 - T2 - T3 - T4 | 1 (5.8)  4 (23.5)  8 (47.1)  2 (11.8)  2 (11.8) |
| Pathological N (pN) stage*, n (%)   - N0 - N1 - N2 | 6 (35.3)  7 (41.2)  4 (23.5) |
| R status, n (%)   - R0 - R1 | 11 (64.7)  6 (35.3) |
| Neoadjuvant chemotherapy, n (%)   - FOLFIRINOX - Gemcitabine/nab-paclitaxel | 7 (41.2)  10 (58.8) |
| Neoadjuvant RAdAR, n (%)   - SAbR - HART | 15 (88.3)  2 (11.7) |

FOLFIRINOX, fluorouracil, leucovorin, oxaliplatin; RAdAR, Risk Adapted Ablative Radiotherapy; SAbR, stereotactic ablative radiation therapy; HART, hypofractionated ablative radiotherapy.

* Per the AJCC staging system, eighth edition.
